# Queues, crowds, and angry mobs: Face identification under distraction in a virtual airport

**DOI:** 10.1177/17470218231203939

**Published:** 2023-11-08

**Authors:** Matthew C Fysh, Edward Baker, Jodie Rockett, John Allen, Cade McCall, A Mike Burton, Markus Bindemann

**Affiliations:** 1 School of Psychology, University of Kent, Canterbury, UK; 2 Tavistock and Portman NHS Foundation Trust, London, UK; 3 Department of Psychology, York University, York, UK; 4 Faculty of Society & Design, Bond University, Robina, QLD, Australia

**Keywords:** Unfamiliar face matching, virtual reality, attention, airport, avatar

## Abstract

In visual environments, selective attention must be employed to focus on task-relevant stimuli. A key question here concerns the extent to which other stimuli within the visual field influence target processing. In this study, we ask whether face identity matching is subject to similar effects from irrelevant stimuli in the visual field, specifically task-irrelevant people. Although most previous studies rely on highly controlled face and body stimuli presented in isolation, here we use a more realistic environment. Participants take the role of passport officers and must match a person’s face to their photo-ID, while other people appear in the background, waiting to be processed. Presenting an interactive virtual environment on screen (Experiments 1 and 2) or in immersive VR (Experiment 3), we generally found no evidence for distraction from background people on face-matching accuracy. However, when immersed in VR, an angry crowd in the background delayed matching speed while not affecting accuracy. We discuss the theoretical implications of these results and their potential importance in practical settings.

## Introduction

The visual environment provides a constant stream of rich sensory input, from which information must be selected for further processing, based on its relevance to current task priorities. A central issue here is the extent to which task-irrelevant stimuli from the visual field are processed and can interfere with observers’ goals. An important class of social stimulus that appears to undergo considerable processing, even when completely irrelevant to the observer’s prescribed task, is other people. Their faces and bodies are detected rapidly in the visual field (Bindemann & Lewis, 2013; Crouzet et al., 2010; Crouzet & Thorpe, 2011; Downing et al., 2004) and can capture (Hershler et al., 2010; Hershler & Hochstein, 2005; Langton et al., 2008; Theeuwes & Van der Stigchel, 2006) and retain visual attention (Bindemann, Burton, Hooge, et al., 2005; Ro et al., 2001). Moreover, studies of target-distractor interference demonstrate that task-irrelevant human stimuli are difficult to ignore and can undergo considerable processing. For example, when distractor faces are presented alongside non-face targets, this can reduce the speed and accuracy of target classification (Bindemann, Burton, & Jenkins, 2005; Jenkins et al., 2005; Young et al., 1986).

These studies have focussed on highly controlled laboratory methods, featuring cropped faces, static displays, and short display times (e.g., 200 ms). How the perception of people proceeds under more complex viewing conditions is less clear. In this study, we examine this question with a scenario that is of everyday importance, but which also provides an interesting contrast to the existing laboratory work, by studying person identification at passport control. Airports are highly populated places in which many people are routinely present, yet within which the important one-to-one task of passport control must also proceed. Typically, this requires the comparison of a photo-ID with the face of its bearer to confirm their identity. In the laboratory, the accuracy of this task has been studied with image-matching paradigms, in which observers are asked to compare pairs of face photos to determine whether these show the same person or different people. This work shows that face matching is surprisingly difficult, with average error rates of up to 35%, depending on the stimuli at hand (e.g., Burton et al., 2010; Fysh & Bindemann, 2018; White et al., 2022).

However, a disconnect exists between laboratory studies of face matching and its real-world application. In contrast to highly controlled laboratory paradigms, person identification at airports involves comparisons between a live person and their photo-ID, and this typically occurs in the presence of other people waiting in the background. Whether these additional person stimuli affect target identification is not known. As a consequence, it is difficult to resolve whether research findings from the laboratory on attention capture and interference by human stimuli generalise to such real-world problems.

One reason for this disconnect is that such scenarios can be difficult to study with the existing laboratory paradigms that are typically employed in this domain. We have recently developed a new approach to address such questions, by using virtual reality (VR) to study face matching in a simulated airport environment (Tummon et al., 2019, 2020). We have populated this airport with photo-realistic avatars, constructed from detailed 3D scans of real people. In our validation work, we have shown that these avatars are recognised as accurately as photographs of the same people and also produce a similarity-space that corresponds with the people upon which they are based (Fysh et al., 2022). Moreover, we have demonstrated a correspondence between the identification of these avatars from photo-ID and established laboratory tests of face matching (Bindemann et al., 2022).^1^ This virtual airport setting therefore provides a platform to study whether task-irrelevant people influence face processing under conditions that provide a closer approximation to real-world scenarios while retaining control over stimulus presentation and behaviour measurement.

In the experiments reported here, participants take on the role of passport control officers, by comparing avatar travellers in the airport to passport-style face photographs in identity documents. The main question of interest is whether this task is affected by other task-irrelevant people in the visual field. For this purpose, the positioning and behaviour of avatars in the surrounding visual background were manipulated. These avatars either formed an orderly queue at passport control, minimising their visibility behind the target person, or were presented as a crowd that was spread out across the background to maximise their visibility. Human motion and body language can also capture visual attention (e.g., Williams et al., 2019). Therefore, the body language of the background avatars was also manipulated to simulate a patiently waiting crowd or a crowd embodying a more restless, agitated state. If task-irrelevant people in the background compete for attention under these conditions, then the virtual crowd should draw cognitive resources away and impair face matching of the target avatars to their photo-IDs. Moreover, these effects might be exacerbated by a restless avatar crowd.

## Experiment 1

This experiment investigated whether face matching is affected by the presence of other people. Using a VR airport paradigm, observers matched avatars of real people to digital face photographs in photo-ID cards. This face-matching task was conducted against a background of avatars that were presented as (i) an orderly queue to be minimally visible, (ii) a spread-out crowd, or (iii) an agitated crowd, in which additional body motion was added to increase distractibility.

## Method

### Participants

Ninety participants (52 females, 38 males) with a mean age of 37 years (*SD* = 9.3) were recruited for this study from *Prolific Academic* in exchange for a small fee and were divided evenly across the three conditions. Our sample size was guided by recent work of person identification in VR that employed similar statistical designs (Tummon et al., 2020), and a post hoc power analysis conducted in G*Power (Version 3.1.9.7; Faul et al., 2009) reflected that our sample size was sufficiently powerful to detect an effect of ƞ_p_^2^ = .08 with 1−β = .82 with a conventional *alpha* threshold of *p* = .05. At the time of testing, all participants were residing in the UK and were native English speakers. This study received full ethical approval from the School of Psychology ethics committee and was conducted in accordance with the ethical guidelines stipulated by the British Psychological Association.

### Materials

Stimuli for this experiment consisted of 80 avatar-photo pairings (40 match and 40 mismatch). The avatars were constructed using 3D face scans of real people that were acquired with an Artec Eva 3D Scanner. These face scans were combined with bodies created in Fuse Character Creator software (Version 1.3) and animated for movement using Mixamo auto-rigging software (for full details of this avatar construction process and its validation, see Fysh et al., 2022). To create identity matches, 40 of these avatars were paired with a digital photograph of their real-life counterpart that was taken on the same day that the 3D facial scan was acquired. To create identity mismatches, the remaining 40 avatars were paired by one of the experimenters with a photograph of a different person who was matched for gender and approximate age and judged to be broadly similar in appearance (see Fysh et al., 2022). Photographs were embedded in a passport-style frame and depicted the target identity against a plain background and under even lighting while bearing a neutral expression (see Bindemann et al., 2022). Each image was presented at a size of 200 (w) × 257 (h) pixels.

**Figure 1.**
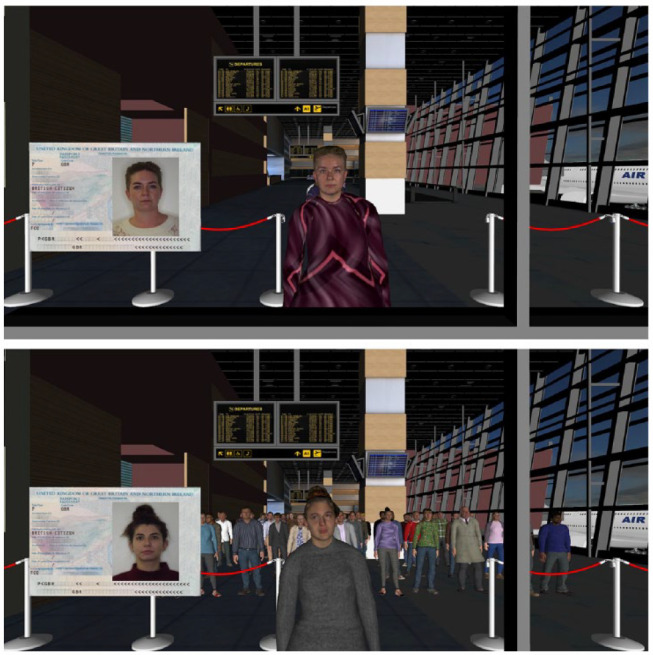
Example match trial from the queue condition (top) and a mismatch trial from the crowd condition (bottom) in Experiment 1, shown from the participants’ perspective. The agitated crowd condition was identical to the regular crowd condition, except that the animation speed was increased to convey restless behaviour.

In the experiments, avatars were presented in the context of a 3D virtual airport hall (https://www.turbosquid.com/3d-models/airport-departures-lounge-3d-model/626226) that was built using 3DS Max (see Tummon et al., 2019), and which was rendered using *Vizard 6* software. In the *queue* condition, the avatars were presented in an orderly queue arranged in a single file, waiting to be processed (see Figure 1). In the *crowd* condition, avatars formed a spread-out forward-facing crowd. In both of these conditions, waiting avatars were programmed to look around and shift their stance occasionally while they were waiting to be processed. The *agitated crowd* condition was identical to the other crowd condition, except that idle animation speed was increased by a scale factor of two for one-half of the avatars and by a factor of three for the remaining proportion, to convey a sense of restlessness (see Tummon et al., 2020). Upon arriving at the passport control kiosk, avatars in this condition reverted to standard speed. In all conditions, the avatars’ animation cycles were offset to prevent synchronised movements. The number of avatars waiting to be processed depleted over time as observers proceeded through the task.

### Procedure

This experiment was conducted during the global COVID-19 pandemic, which prevented in-person testing. Participants were therefore tested remotely, whereby the experimenter shared their screen with participants via Zoom telecommunications software. Participants were provided with a view of the airport from the perspective of a passport control booth. Avatars approached the booth individually and were presented alongside a photo-ID (see Figure 1). Participants were instructed to decide whether each avatar-photo pairing depicted the same person or two different people by verbally stating “same” or “different.” Participants’ vocal responses were then manually entered by the experimenter, thereby initiating the next trial. Upon registration of a response, the current avatar moved past the passport control desk, the next avatar moved forward to the desk, and the photo-ID was updated to the next person. Each participant provided identification decisions for 80 avatar-photo pairings (40 match and 40 mismatch). The queue, crowd, and agitated crowd conditions were administered on a between-subjects basis.

## Results^2^

### Accuracy

**Figure 2.**
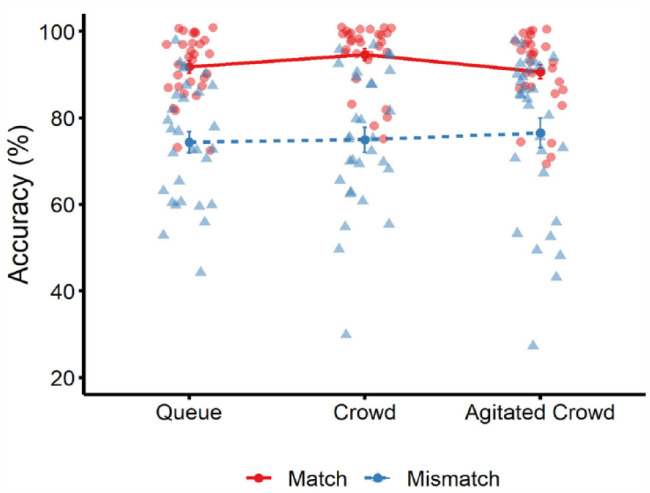
Mean percentage accuracy for match trials (solid red line) and mismatch trials (dotted blue line) across the three conditions in Experiment 1. Circle markers correspond to data points on match trials, and triangle markers correspond to data points on mismatch trials. Error bars denote standard error of the mean.

The percentage of correct face-matching decisions was calculated for all conditions. The cross-subject means of this data are illustrated in Figure 2. To analyse these data, a 3 (group type: queue, crowd, agitated crowd) × 2 (trial type: match vs. mismatch) mixed-factor analysis of variance (ANOVA) was conducted to explore the probability of observing significant effects under the null model. Bayesian analyses were also run in JASP using default parameters (JASP Team, 2022) to indicate the relative strength of evidence in favour of the alternative over the null model (Van Doorn et al., 2021).

These analyses revealed an effect of trial type, *F*(1, 87) = 62.76, *p* < .001, ƞ_p_^2^ = .42, BF_10_ = 5.177 × 10^13^, due to higher accuracy on match than mismatch trials. However, there was no effect of group type, *F*(2, 87) = 0.40, *p* = .67, ƞ_p_^2^ = .01, BF_10_ = 0.06. In addition, the interaction between factors was not significant, *F*(2, 87) = 0.56, *p* = .57, ƞ_p_^2^ = .01, BF_10_ = 0.05.

### Sensitivity and criterion

Accuracy was also converted to loglinear signal detection measures of sensitivity (*dʹ*) and criterion (*c*) to measure overall performance and response bias (Hautus, 1995; Stanislaw & Todorov, 1999). Sensitivity refers to an individual’s ability to distinguish between two similar stimulus classes (e.g., matches and mismatches in the current context), with higher values indicating better ability. Criterion captures the tendency to respond in a particular way. In the current experiments, a negative criterion value indicates a bias to make more match than mismatch responses.

In this experiment, a between-subjects ANOVA showed that sensitivity was comparable across the queue (*M* = 2.19, *SD* = 0.52), crowd (*M* = 2.48, *SD* = 0.67), and agitated crowd conditions (*M* = 2.24, *SD* = 0.62), *F*(2, 87) = 1.95, *p* = .148, ƞ_p_^2^ = 0.04, BF_10_ = 0.47. Criterion was also similar across the queue (*M* = −0.39, *SD* = 0.41), crowd (*M* = −0.50, *SD* = 0.42), and agitated crowd conditions (*M* = −0.30, *SD* = 0.48), *F*(2, 87) = 1.47, *p* = .235, ƞ_p_^2^ = .03, BF_10_ = 0.32. One-sample *t*-tests showed that criterion was reliably below zero in all three conditions, queue: *t*(29) = 5.18, *p* < .001; *d* = 0.95, BF_10_ = 142 × 10^2^; crowd: *t*(29) = 6.54, *p* < .001, *d* = 1.20, BF_10_ = 460 × 10^2^; agitated crowd: *t*(29) = 3.46, *p* = .002, *d* = 0.63, BF_10_ = 20.70, indicating a consistent bias to classify avatar-face pairings as identity matches.

### Discussion

This experiment investigated face-matching accuracy in a virtual airport while avatars in the background waited to be processed in a queue, a crowd, or an agitated crowd. Across the three conditions, performance was comparable in terms of percentage accuracy, sensitivity, and criterion. These findings provide initial evidence that the presence of task-irrelevant avatars does not impair face-matching accuracy in an airport setting. The absence of such effects is surprising considering that faces and bodies are detected rapidly in the visual periphery (Bindemann & Lewis, 2013; Crouzet et al., 2010; Crouzet & Thorpe, 2011; Downing et al., 2004), can be a powerful draw for visual attention (Langton et al., 2008; Theeuwes & Van der Stigchel, 2006), and can be difficult to ignore even when they are task-irrelevant (Bindemann, Burton, et al., 2005; Jenkins et al., 2005). Moreover, although body language can also capture visual attention (Williams et al., 2019), such an effect was not evident when a crowd of avatars behind the target exhibited agitated language. Thus, these data suggest that the task-irrelevant avatars did not influence the identification of avatar targets in a virtual airport setting.

It is possible, however, that any distracting effects from the background avatars were suppressed by high accuracy on match trials and a general bias to make match responses, which was observed across conditions. This bias converges with other studies, which suggest that the contextual effects of passport control increase the tendency to classify faces as identity matches (see, e.g., Bindemann et al., 2022; Feng & Burton, 2019; McCaffery & Burton, 2016). In addition, the body language manipulation in the agitated crowd condition may have been too subtle to engage their attention. This was investigated further in Experiment 2.

## Experiment 2

In this experiment, task difficulty was increased by pairing a person’s avatar with a photograph that was taken a year after their 3D scan was acquired (see, e.g., Bindemann & Sandford, 2011; Fysh & Bindemann, 2018; Jenkins et al., 2011; Megreya et al., 2013). In addition, the neutral crowd condition from Experiment 1 was replaced with an angry mob condition, in which avatars made aggressive gestures towards the participant while waiting to be processed. There is evidence that threatening stimuli interfere with target selection (Schmidt et al., 2015) and capture and hold visual attention (Koster et al., 2004, but see Quinlan, 2013). Therefore, an angry mob of avatars might draw attention away from the identification target with greater potency than an agitated crowd. In addition, angry crowd sounds were added to the angry mob condition, overlaid on ambient airport sounds. The detection of angry voices is rapid and automatic (Burra et al., 2019; Gädeke et al., 2013; Sander et al., 2005; Sauter & Eimer, 2010), and can exert cross-modal effects to capture visual attention (Brosch et al., 2009). By combining visual and auditory information in this way, we sought to maximise the distracting influence of the crowd in the angry mob condition. Therefore, we expected accuracy to be highest in the queue condition and lowest in the angry mob condition. Performance with the agitated crowd context served as a benchmark of whether the comparable accuracy rates across the conditions of Experiment 1 arose due to low stimulus difficulty or because the crowd manipulation was ineffective.

## Method

### Participants

Ninety people (35 males, 55 females) with a mean age of 35.9 years (*SD* = 9.4) were recruited from *Prolific Academic* to participate in this study in exchange for a small fee. These participants were randomly assigned to one of three experimental conditions (*N* = 30 per condition). At the time of testing, all participants were residing in the UK and were native English speakers. None had participated in Experiment 1.

### Stimuli and procedure

The stimuli, airport hall and procedure were identical to Experiment 1, except for the following changes. First, the same-day laboratory photographs were replaced with self-portrait photographs (i.e., “*selfies*”) that were provided by participants 11.1 months (*SD* = 1.4) after their scan date, and which depicted the subject bearing a neutral expression under good lighting and against a plain background. For five of the original subjects whose face scans were used for avatar construction, such images could not be obtained. These were therefore replaced with five new avatar-photo-ID pairings. However, the total number of matches and mismatches remained consistent with that of Experiment 1.

**Figure 3.**
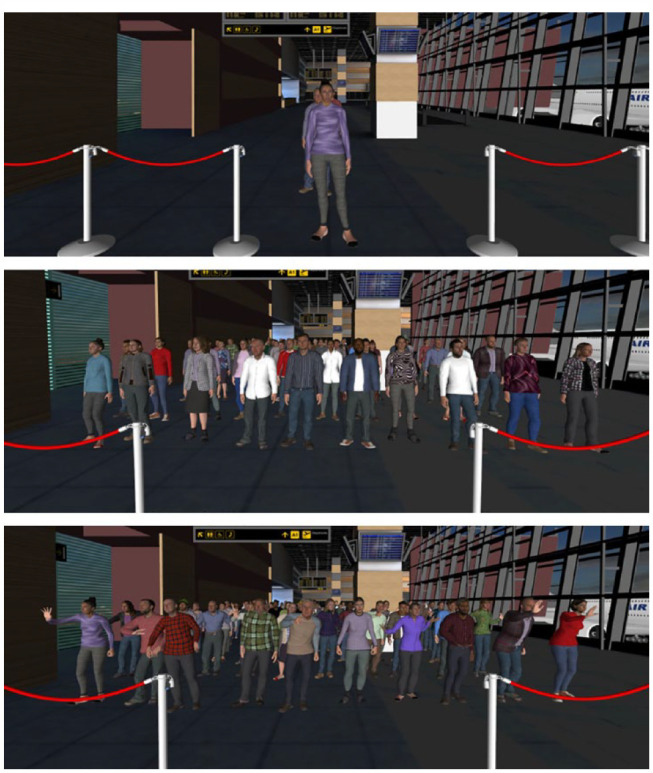
Depictions of the queue (top), agitated crowd (middle), and angry mob (bottom) conditions in Experiment 2.

The second modification consisted of the angry mob condition, whereby the avatars displayed visible aggravation while waiting to be processed, exhibiting behaviours such as finger pointing, hand gesturing, and leaning forwards (for an illustration, see Figure 3). Upon reaching the passport control desk, avatars reverted to the standard idle mode exhibited in the queue conditions. In this condition, angry crowd sound effects^3^ were also administered throughout the experiment, in combination with ambient airport noises.^4^ No sounds were provided in the queue and agitated crowd conditions.

## Results

### Accuracy

**Figure 4.**
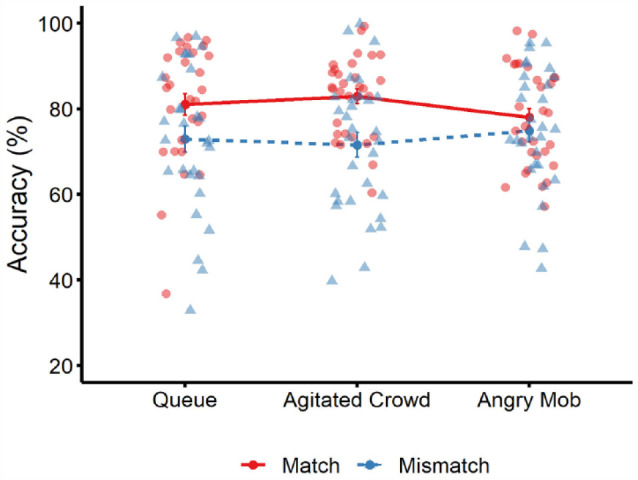
Mean percentage accuracy for match trials (solid red line) and mismatch trials (dotted blue line) across the three conditions in Experiment 2. Circle markers correspond to data points on match trials, and triangle markers correspond to data points on mismatch trials. Error bars denote standard error of the mean.

The cross-subject means of match and mismatch accuracy for all conditions are visualised in Figure 4. A 3 (group type: queue, agitated crowd, angry mob) × 2 (trial type: match vs. mismatch) mixed-factor ANOVA revealed a main effect of trial type, *F*(1, 87) = 8.85, *p* = .004, ƞ_p_^2^ = .09, BF_10_ = 66.16, due to higher accuracy on match trials compared to mismatch trials. A main effect of group type, *F*(2, 87) = 0.12, *p* = .888, ƞ_p_^2^ < .01, BF_10_ = 0.06, and an interaction between factors were not found, *F*(2, 87) = 0.88, *p* = .418, ƞ_p_^2^ = .02, BF_10_ = 0.08.

### Sensitivity and criterion

Sensitivity and criterion were analysed via separate one-way ANOVAs. This showed that sensitivity was comparable for the queue (*M* = 1.63, *SD* = 0.48), agitated crowd (*M* = 1.63, *SD* = 0.53), and angry mob condition (*M* = 1.54, *SD* = 0.40), *F*(2, 87) = 0.38, *p* = .683, ƞ_p_^2^ = .01, BF_10_ = 0.14. Criterion also did not vary significantly across the queue (*M* = −0.13, *SD* = 0.47), agitated crowd (*M* = −0.17, *SD* = 0.41), and angry mob condition (*M* = −0.05, *SD* = 0.41), *F*(2, 87) = 0.65, *p* = .527, ƞ_p_^2^ = .02, BF_10_ = 0.17. Finally, one-sample *t*-tests revealed that criterion was comparable to zero in the queue condition, *t*(29) = 1.49, *p* = .147, *d* = 0.27, BF_10_ = 0.53, and the angry mob condition, *t*(29) = 0.66, *p* = .516, *d* = 0.12, BF_10_ = 0.24 but was below zero with the agitated crowd, *t*(29) = 2.30, *p* = .029, *d* = 0.42, BF_10_ = 1.86, indicating a bias in this condition to classify faces as identity matches.

### Discussion

This experiment replicates the main findings of Experiment 1. Despite the increased difficulty of match trials and the addition of an angry crowd condition with more exaggerated body language, accuracy and sensitivity were similar across group conditions, indicating that face matching at the virtual airport is not affected by the presence of other avatars in the visual field. It is possible, however, that the behaviour displayed by the angry mob failed to exert its full effect due to the 2D onscreen presentation of the stimuli. To address this possibility and to maximise the generalisability of our findings to the real world, we conducted a final experiment in which the queue and angry mob conditions were repeated in immersive VR.

## Experiment 3

The previous experiments were conducted with non-immersive VR, whereby the airport was presented on a computer screen. In Experiment 3, this was replaced with a tracked head-mounted display (HMD) that responds to user movement. The application of such headsets invokes a higher sense of person presence and immersion than non-immersive VR (see Pallavicini et al., 2019; Pallavicini & Pepe, 2019). To provide a stronger test for the claim that people in the background do not influence face matching, this experiment therefore replicated the queue and angry mob conditions of Experiment 2 with such immersive VR. This move to VR makes it possible to determine whether the absence of an effect in Experiments 1 and 2 occurred because the general feeling of realism was not adequately captured by the remote delivery of the procedure. Given that we are primarily interested in how irrelevant people in the real world might impact face identity matching, studying this problem in VR is an essential step towards understanding whether performance is influenced by the presence and behaviour of queues and crowds.

Immersive VR also allows for the recording of participants’ responses, using handheld controllers. This provides response times as an additional measure in Experiment 3, to determine whether the queue and angry mob condition exert dissociable effects on target processing during face matching. Thus, response times to the targets should be slower if an angry mob is more distracting than the orderly queue.

## Method

### Participants, stimuli, and procedure

Twenty students were recruited for each condition of this experiment, for a combined total of 40 participants (15 males, 25 females) from the University of Kent, with a mean age of 21 years (*SD* = 5.1). The stimuli and procedure for this experiment were identical to Experiment 2, except that only the queue and angry mob conditions were retained, and the study was administered in immersive VR with an HTC Vive HMD. Ambient airport noises were played through headphones in both experimental conditions, in addition to the angry crowd sound effects in the mob condition. Participants classified stimuli as matches and mismatches via two buttons on handheld controllers.

### Results

#### Accuracy

**Figure 5.**
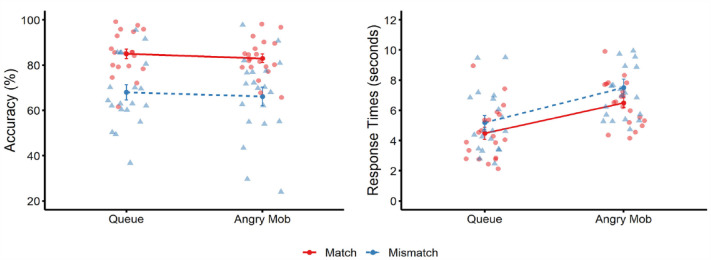
Mean percentage accuracy (left) and mean correct response times (right) for match trials (solid red line) and mismatch trials (dotted blue line) across the two conditions in Experiment 3. Circle markers correspond to data points on match trials, and triangle markers correspond to data points on mismatch trials. Error bars denote standard error of the mean.

Matching accuracy for both person conditions is illustrated in Figure 5. A 2 (group type: queue vs. angry mob) × 2 (trial type: match vs. mismatch) mixed-model ANOVA revealed a main effect of trial type, *F*(1, 38) = 27.18, *p* < .001, ƞ_p_^2^ = .42, BF_10_ = 164 × 10^3^, due to higher accuracy on match than mismatch trials. There was no main effect of group type, *F*(1, 38) = 0.49, *p* = .488, ƞ_p_^2^ = .01, BF_10_ = 0.28, or an interaction, *F*(1, 38) < 0.01, *p* = .985, ƞ_p_^2^ = .00, BF_10_ = 0.29.

#### Sensitivity and criterion

For sensitivity, an independent-samples *t*-test revealed no difference between the queue (*M* = 1.60, *SD* = 0.57) and the mob conditions (*M* = 1.44, *SD* = 0.60), *t*(38) = .88, *p* = .384, *d* = .28, BF_10_ = 0.42. Criterion was also comparable between the queue (*M* = −0.29, *SD* = 0.37) and mob conditions (*M* = −0.26, *SD* = 0.37), *t*(38) = .27, *p* = .788, *d* = .09, BF_10_ = 0.32, and was significantly below zero with queue, *t*(19) = 3.55, *p* = .002, *d* = .79, BF_10_ = 19.01, and angry mob displays, *t*(19) = 3.19, *p* = .005, *d* = .71, BF_10_ = 9.50.

#### Response times

Mean correct response times are also depicted in Figure 5. As this was not a speeded task, there was no outlier processing or trimming of RTs. A 2 (group type) × 2 (trial type) mixed-factor ANOVA revealed a main effect of trial type, *F*(1, 38) = 15.49, *p* < .001, ƞ_p_^2^ = .29, BF_10_ = 60.86, due to faster responses on match than mismatch trials. A main effect of group type was also found, *F*(1, 38) = 13.26, *p* < .001, ƞ_p_^2^ = .26, BF_10_ = 28.43, as faces were matched more slowly in the angry mob than in the queue condition. An interaction between factors was not found, *F*(1, 38) = 0.42, *p* = .520, ƞ_p_^2^ = .01, BF_10_ = 1.31.

In light of the effect of group type on response times, we also examined whether this effect changed over time, with the depletion of the crowd over the course of the experiment, by comparing response time for the first and second half of trials^5^. A 2 (group type) × 2 (trial type) × 2 (time) mixed-factor ANOVA of these data did not show a main effect of time, *F*(1, 38) = 1.84, *p* = .183, ƞ_p_^2^ = .05, BF_10_ = 11.58, or a three-way interaction, *F*(1, 38) = 1.48, *p* = .232, ƞ_p_^2^ = .04, BF_10_ = 0.44. However, an interaction of time and group was found, *F*(1, 38) = 9.31, *p* = .004, ƞ_p_^2^ = .20, BF_10_ = 43.12, whereby response times were slower in the first half of the experiment than the second half in the queue condition (*M* = 5,439 ms, *SD* = 2,314 vs. *M* = 4,282 ms, *SD* = 1,605), *t*(19) = 3.47, *p* = .003, *d* = .78, BF_10_ = 16.15. In contrast, no effect of time was observed in the angry mob condition (*M* = 6,818 ms, *SD* = 1,614 vs. *M* = 7,264 ms, *SD* = 2,624), *t*(19) = 1.10, *p* = .286, *d* = .25, BF_10_ = 0.39.

### Discussion

This experiment employed immersive VR to compare face matching in the presence of a queue or an angry mob. Consistent with laboratory studies of target-distractor processing, an effect was observed in response times (Bindemann, Burton, et al., 2005; Jenkins et al., 2005), whereby observers were slower to classify faces in the mob than in the queue condition. This suggests that the presence of additional people in the visual field does engage observers’ attention, and draws these resources away from the target faces that are of primary relevance. However, as in Experiments 1 and 2, no evidence of interference on face-matching accuracy or sensitivity was found for the queue and crowd conditions, which indicates that the difference in response time between conditions did not translate into these measures.

## General discussion

This study investigated whether the processing of target faces is affected by concurrently presented people in the background. In a departure from previous research, which has investigated this question with simplistic laboratory displays and short display times (e.g., Bindemann, Burton, et al., 2005; Jenkins et al., 2005), the present experiments were designed to explore whether such distraction is observed under conditions of greater complexity and ecological validity. For this purpose, we employed a VR airport, in which participants act as passport controllers by matching photo-IDs to the faces of avatar travellers. Distraction by task-irrelevant faces was then assessed by populating the airport background with an orderly queue of travellers, which were aligned so that their visibility was minimised to participants. This was contrasted with crowd conditions, in which the avatars were distributed across the airport to maximise visibility. In some crowd conditions, the avatars also exhibited agitated and angry body language and angry crowd sounds, to further increase their distracting influence.

Across the three experiments, an effect of the crowd manipulation was only observed in response times in Experiment 3, whereby face-matching decisions were made more slowly in the presence of an angry mob than a passenger queue when these conditions were administered in immersive VR. This condition was designed to maximise distractibility by combining the influences of task-irrelevant faces, bodies, body language, and crowd sounds. At this point, we cannot partial out the relative contribution of each of these factors. Importantly, however, face-matching accuracy, sensitivity, and criterion were unaffected in all experiments by the formation of avatars into queues and crowds, as well as the behaviour exhibited by crowds and the ambient sounds. We interpret this pattern of effects as evidence that observers are distracted by the presence of the angry mob, which can divert attention from the face-matching task and increase response times. A number of laboratory studies already suggest that people have distracting powers and can undergo considerable visual processing even when they are task-irrelevant (e.g., Bindemann, Burton, et al., 2005; Jenkins et al., 2005; Langton et al., 2008; Theeuwes & Van der Stigchel, 2006). However, observers can also override such influences endogenously to re-assert focus on a target (Bindemann Burton, et al., 2007). The observation of a response latency effect in Experiment 3, but no effect in accuracy suggests that observers were distracted by the angry mob but were also able to re-assert control and endogenously shift attention to the avatar-photo pairings, to complete task-relevant face processing in the matching task.

A key difference between the current VR paradigm and previous laboratory studies is that the airport allows for serial processing of targets and distractors (queues and crowds), whereas the millisecond display times of lab studies are designed to limit such opportunities. This emphasises the importance of testing the correspondence between laboratory phenomena and more complex settings. Within the context of passport control in the real world, the current findings imply that passport officers might be slower to process an agitated crowd of travellers, but that this is unlikely to have a knock-on effect on identification accuracy. In future studies, the extent to which crowds affect face matching could be examined further by introducing additional variables that might be at play in airport settings. For example, in contrast to the current experiments, where identity mismatches and matches were presented with equal frequency, mismatches occur only infrequently in real-world passport control settings. Infrequent targets are more likely to be missed by observers (Wolfe et al., 2005), and some similar effects have been observed in face matching (Papesh & Goldinger, 2014; Weatherford et al., 2020, 2021). If such factors interact with crowding in the virtual airport, then this might give rise to a different pattern of effects than was observed here.

Reduced mismatch frequency can also lead to an increase in match responses in face matching (Papesh & Goldinger, 2014; Weatherford et al., 2020, 2021). Although the queue, crowd, and mob manipulations failed to elicit accuracy differences in all experiments, a response bias to classify avatars and photo-ID pairings as identity matches was also observed consistently in the current experiments. This bias in face matching has been observed in similar behaviourally relevant contexts (see, Bindemann et al., 2022; Feng & Burton, 2019; McCaffery & Burton, 2016; Robertson & Burton, 2021), from embedding faces in passport-style photo frames (McCaffery & Burton, 2016) to matching avatars to photographs in VR (Bindemann et al., 2022). The persistent observation of this bias in the current work further reflects that the contextual power of an airport is sufficient to reduce the capacity of novice participants to identify pairs of faces that belong to different people (i.e., mismatches).

The real-world implications of this finding may be considerable. A key aim of passport control is to detect identity impostors, who travel with the legitimate photo-ID documents of someone with similar appearance to evade detection (Stevens, 2021). The match bias implies that the detection of such real-world mismatches should be even more difficult in airport settings than laboratory experiments with static face pairs would suggest. As VR is becoming more prevalent in the study of person identification to bridge the knowledge gap between laboratory paradigms and complex real-world settings, understanding the cause of this match bias, and the wider impact of real-world variables on face-matching accuracy, is moving into reach.

## Notes

1. We note that this task requires identity matching across a change in format, from avatar to photograph. Studies of face matching typically also pair images captured with different devices (see, e.g., Burton et al., 2010; Fysh & Bindemann, 2018; Megreya & Burton, 2006), which can introduce meaningful differences between images (see Noyes & Jenkins, 2017), or compare video-to-photo (e.g., Bruce et al., 1999), live-to-photo (e.g., Kemp et al., 1997; Megreya & Burton, 2008), or CCTV-to-live matching (Davis & Valentine, 2009). Thus, format-based differences between to-be-compared stimuli are a common characteristic of face-matching studies and not unique to the virtual airport paradigm employed here.

2. The data for all experiments reported here can be accessed at https://osf.io/hve39/?view_only=abcd869d9f314172b11e71af0c669d28

3.
https://www.youtube.com/watch?v=40mBBPZKf0I&ab_channel=SoundEffectDatabase

4.
https://www.youtube.com/watch?v=8trgESN1Eks&t=52s&ab_channel=MotionArray

5. For completeness, the data are available for all experiments reported here at https://osf.io/hve39/?view_only=abcd869d9f314172b11e71af0c669d28
